# Uncorrected refractive error and associated factors among primary school children in Debre Markos District, Northwest Ethiopia

**DOI:** 10.1186/1471-2415-14-95

**Published:** 2014-07-29

**Authors:** Sintayehu Aweke Sewunet, Kassahun Ketema Aredo, Molla Gedefew

**Affiliations:** 1 Debre Markos referral hospital, East Gojjam Zone, Amhara Regional State, Debre Markos, Ethiopia; 2 Department of Public Health, Debre Markos University, College of Medicine and Health Sciences, PO Box: 269, Debre Markos, Ethiopia; 3 Department of medicine, Gamby College of Medical Sciences, Bahir Dar, Ethiopia

**Keywords:** Refractive error, Myopia, Students, School, Children

## Abstract

**Background:**

Uncorrected Refractive Error is one of the leading cause amblyopia that exposes children to poor school performance. It refrain them from productive working lives resulting in severe economic and social loses in their latter adulthood lives. The objective of the study was to assess the prevalence of uncorrected refractive error and its associated factors among school children in Debre Markos District.

**Method:**

A cross section study design was employed. Four hundred thirty two students were randomly selected using a multistage stratified sampling technique. The data were collected by trained ophthalmic nurses through interview, structured questionnaires and physical examinations. Snellens visual acuity measurement chart was used to identify the visual acuity of students. Students with visual acuity less than 6/12 had undergone further examination using auto refractor and cross-checked using spherical and cylindrical lenses. The data were entered into epi data statistical software version 3.1 and analyzed by SPSS version 20. The statistical significance was set at α ≤ 0.05. Descriptive, bivariate and multivariate analyses were done using odds ratios with 95% confidence interval.

**Result:**

Out of 432 students selected for the study, 420 (97.2%) were in the age group 7–15 years. The mean age was 12 ± 2.1SD. Overall prevalence of refractive error was 43 (10.2%). Myopia was found among the most dominant 5.47% followed by astigmatism 1.9% and hyperopia 1.4% in both sexes. Female sex (AOR: 3.96, 95% CI: 1.55-10.09), higher grade level (AOR: 4.82, 95% CI: 1.98-11.47) and using computers regularly (AOR: 4.53, 95% CI: 1.58-12.96) were significantly associated with refractive error.

**Conclusion:**

The burden of uncorrected refractive errors is high among primary schools children. Myopia was common in both sexes. The potential risk factors were sex, regular use of computers and higher grade level of students. Hence, school health programs should work on health information dissemination and eye health care services provision.

## Background

Refractive error is a state in which optical system of the eye fails to adjust to bring parallel rays of light to focus on proper place (fovea). It is obvious that “without appropriate optical correction, millions of children are losing educational opportunities and adults are excluded from productive working lives, facing severe economic and social consequences. Individuals and families are pushed into a cycle of deepening poverty because of this health problem” [1,2].

Uncorrected refractive error is the leading cause of eye problem worldwide and the second cause of blindness [3]. Worldwide, there are about 2.3 billion people have refractive error. Out of these peoples, only 1.8 billion have access to eye health care services which are affordable correction. Children are more vulnerable group, because uncorrected refractive error can result in to a dramatic impact on learning process and educational capacity [4]. Most of the children with such diseases are apparent and hence screening helps in early detections and correction [5].

This eye problem is a reason for a number of blind for years by a person than most other causes if not corrected early. The blindness due to refractive error can be resulted in an average of 30 years which 5 years in case of untreated cataract [6].

Special attention has to be given to school age because it is the age at which refractive error begins. The prevalence of myopia is less than 2% before 7 or 8 years but increases with age and reaches 20% at 15 year. The potential risk factors for myopia were family history and near work during childhood, and time spent outdoors [6,7].

Most refractive errors can be managed by early refractive correction. If it cannot treated in childhood may come up with amblyopia, resulting in blindness. The correction can be done by spectacles, contact lenses, or refractive surgery. The most commonly used correction method is spectacles. Hence, spectacles are treatment for refractive error in developing countries [8].

The situation is worse in Sub-Saharan Africa, including Ethiopia. The level of refractive error in Debre Markos, Ethiopia has not been previously addressed. Lack of awareness of the students towards refractive error in addition to the painless and progressive nature of this disease is believed to be among the causes which let the problem remain undetected or uncorrected. Unavailability of and/or inaccessibility to eye care services are also serious problems in Ethiopian.

Paucity of sufficient information in Ethiopia is another problem which restrains the decision makers at various levels from taking preventive as well as corrective measures. It was with these backgrounds that the researcher determined to investigate the prevalence of uncorrected refractive error of school children in Debre Markos district and identify modifiable risk factors that have paramount importance for the improvement of programs aimed at prevention and control.

## Methods

### Study design

School based cross sectional study was conducted in Debre Markos district, Northwest Ethiopia in March 2013. The district is located about 300 kilo meters from the capital city, Addis Ababa [9]. According to the 2012 East Gojjam Zone administrative office report, the district has a total population of 86,786 within seven kebeles of which children below 15 years, at primary school level, account 18,345, boys 8,325 (45.4%) and girls 10,020 (55.6%).

The district has one public referral hospital which serves East Gojjam zone and other nearby zones; and two private eye clinics institution giving eye care service. There are 23 primary schools excluding the school for the blind. They comprise a total of 11,842 students. Out of these 23 primary schools, 4 are found in rural areas. Eight of them are private owned whereas 15 are public schools (unpublished district education office record). The source populations of the study were school children with the age of 5–16 years living in the district. Public and private schools of the district were randomly selected. Then children, who met the inclusion criteria from the target, were selected as the subjects of the study. The study excluded children with eye injury and with other serious sickness.

### Sampling methods

The sample size was calculated using single population proportion formula by taking into consideration 9.4% prevalence rate of refractive errors among schoolchildren in rural central Ethiopia from previous study [10]. A total of 432 students were selected for the study by applying the formula.

A multistage stratified sampling technique proportional to size was applied. All primary schools were listed according to ownership as public and private. Samples were randomly selected from each group according to their size. Further classification of the selected schools into different grades/standards was made and the selected grades/standards were further classified into randomly selected sections. Finally, sample students were selected from these sections using systematic random sampling.

### Operational definition

• Amblyopia: is a reduced visual capacity in one or both eyes (commonly called lazy eye) in the absence of another specific eye disease.

• Astigmatism: distorted vision resulting from an irregularly curved cornea, the clear covering of the eyeball.

• Hyperopia (farsightedness): difficulty in seeing close objects clearly;

• Myopia (nearsightedness): difficulty in seeing distant objects clearly;

• Presbyopia: Universal difficulty in reading or seeing at arm’s length, due to age.

• Regular use of computers,video or reading: reading/watching at least once a day for not less than 2 hours.

• Visual acuity: Ability to identify letters at a distance of 6 meters.

• Visual impairment: Visual acuity less than 6/12 in the better eye without classes.

### Data collection procedure

The data were collected by ophthalmic professionals, optometrists and ophthalmic nurses with good experience of regional surveys and are working on refractive service.

Thorough interviews were made using structured questionnaires and physical examinations were conducted using Snellens chart at six meters, illumination and ophthalmoscope at school. Students with less than 6/12 had undergone further examination by slit-lamp and correction of their refractive error at the hospital. Students with refractive error underwent refraction using auto refractor and were cross checked subjectively using trial lenses.

The questionnaires were pre–tested and the necessary corrections were made before the actual data collection. A one day training was given to the data collectors. The principal investigators closely supervised the entire data collection processes. The filled out questionnaires and examination results were collected after checking for consistency and completeness on daily base. Double entry of 5% data for checking errors was made.

### Data analysis

Data were entered using epi data statistical software version 3.1 and analyzed using SPSS version 20. Descriptive statistics using tables and graphs was presented. Binary logistic analysis with conditional method calculating odds ratios (OR) and 95% confidence intervals (CI) was used to estimate the association between the dependent variable and independent variables. Statistical significance was set at α. ≤ 0.05.

In an attempt to identify the relative effects of explanatory variables on the outcome variable, hierarchical multivariable analyses was applied. Explanatory variables with P-value <0.2 were entered into the final regression model based on the likelihood ratio for further analyses in two different models.

### Ethical considerations

Ethical approval and clearance was sought from the ethical review board of Debre Markos University, College of Medicine and Health Sciences, Department of Public Health. Support letter and permission was obtained from the district education office and school administrators. The purpose of the study was explained to parents or guardian and written consents were obtained from parents. Verbal consents were also obtained from all eligible students who took part in the study. Students with refractive error underwent full refraction using auto refractor following standard procedure and given prescriptions. Those students with infectious ocular problems were given Tetracycline.

## Result

### Socio-demographic characteristics

A total of 432 students were randomly selected for the study with a response rate of 420 (97.2%). The mean age of respondents was 12.08 ± 2.1SD. Even though, the proportion of students in the school was almost equal, females accounted for 240 (57%) and 375 (81.1%) of the respondents were from urban schools. Most of students 370 (88.1%) were from public school. Concerning grade level of students, 241 (57.3%) were from grades 1–4 whereas the remaining 179 (42.6%) were from grades 5–8 (Table 1).

**Table 1:** Socio demographic characteristics of school children in Debre markos district, March 2013

**Variable**	**Category**	**Frequency(420)**	**Percent**
Age	7-11	194	46.2
	12-15	226	53.8
Sex	Male	181	43.1
	Female	239	56.9
Residence	Urban	345	82.1
	Rural	75	17.9
School by ownership	Public	370	88.1
	Private	50	11.9
Grade level	1-4	241	57.4
	5-8	179	42.6
Family educational status	No read and write	99	7.4
	Read and write	155	37.3
	Primary education	28	6.7
	Secondary education	49	11.7
	Higher education	85	20.4

### Prevalence of refractive error

**Figure 1:**
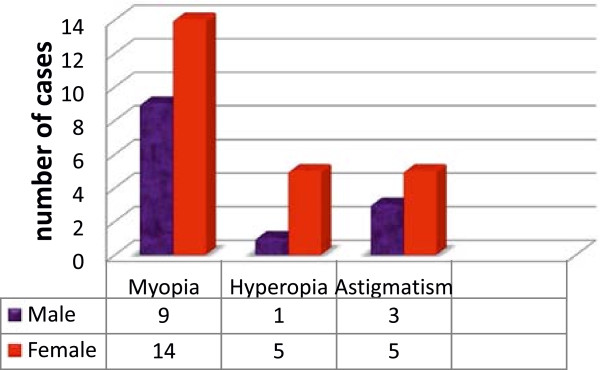
Types of refractive error by sex among school children in Debre Markos district, March 2013.

Forty-nine (11.6%) children were found to have visual impairment. Of these 43 (10.2%) had refractive error. Five (10%) were due to corneal problem where as the remaining 1 (2%) was due to other factors. The prevalence of uncorrected refractive error was 41 (9.5%). Myopia is the leading cause of refractive error 23 (5.47%) followed by astigmatism 8 (1.9%) and hyperopia 1.4% in both sex (Figure 1).

Refractive error was found in 31 (12%) of females whereas, 6.7% were males. Among students with refractive error, only 4 (9.3%) were previously refracted and two were using eye glasses. Students who underwent preschool and school screening for refractive error were 21 (5%).

Twenty three (5.47%) children had their right eyes and 19 (4.5%) their left eyes improved in their visions with pinhole to better than 6/12. Forty-nine (11.6%) of the students were found to have visual impairment in one or both eyes (Table 2).

**Table 2:** Unaided and pinhole Visual acuity of primary school children, Debre Markos district, March 2013

**Unaided Visual acuity**	**Right eye Un aided vision**		**Right eye Pinhole vision**		**Left eye unaided vision**		**Left eye pinhole vision**	
	**No. of students**	**%**	**No. of students**	**%**	**No. of students**	**%**	**No. of students**	**%**
**≥6/12**	371	88.3	393	93.6	396	89.5	415	94
**<6/12-6/18**	8	1.9	15	3.6	29	6.9	20	4.8
**6/18-6/60**	24	5.7	8	1.9	10	2.4	3	0.7
**6/60-3/60**	12	2.9	3	0.7	4	1	1	0.2
**<3/60**	5	1.2	1	0.2	1	0.2	1	0.2

### Factors associated with uncorrected refractive errors

In multiple logistic regression analysis, potential risk factors for uncorrected refractive errors were female sex, higher grade level and using computers regularly. Feeling to have visual problem and difficulty of seeing distant objects were found independently associated with refractive errors. There was high prevalence in urban 38 (11%) than in rural 5 (6.7%) residents but it was not statistically significant.

Females were about 3.9 times (CI: 1.556-10.092) more likely to experience refractive errors than male students. The prevalence of refractive errors in higher grade levels (5–8) was about 4.8 times (95% CI: 1.980-11.474) more likely than in lower grades (1–4). The children who felt having eye problem had 2.8 times (95% CI: 1.050-6.040) more chance than those who did not have the feeling once. Those with difficulty in seeing distant objects were 2.5 more (95% CI: 1.050-6.040) than those who see well. Children using computers regularly were 4.5 times (95% CI: 1.589-12.968) more affected than non-users or irregularly users (Table 3).

**Table 3:** Association of refractive error and potential factors among school children in Debre Markos District, March 2013

**Variable**	**Category**	**Frequency**	**P.value**	**COR**	**95%CI**	**P.value**	**AOR**	**95%CI**
Age	7-11	193	0.013	2.411	1.22-4.832	0.832	1.2	3.079-4.004
	12-15	227		1			1	
Sex	Male	181	0.037	2.099	1.046-4.212	0.004	3.963	1.556-10.092
	Female	239		1			1	
Grade level	1-4	240	0.001	3.135	1.604-6.128	0.001	4.823	1.980-11.474
	5-8	180		1			1	
Using computers regularly	Yes	45	0.027	2.500	1.111-5.625	0.005	4.539	1.589-12.968
	No	375		1			1	

COR-crude odds ratio.

AOR-adjusted odds ratio.

CI-confidence interval.

The uptake of refractive service was 4 (9.3%) and spectacle utilization was 1 (4.6%). The study revealed that 352 (83.8%) of the students had access to electric light for reading. A few children 135 (32%) claimed that the health institution provided eye care service. There were 64 (15.2%) observed ocular morbidities in either eye.

## Discussion

This study revealed that overall prevalence of refractive error was 43 (10.2%) with visual acuity < 6/12 in the better eye. This finding was fairly comparable with a study in Nepal in 2010 [11]. The slight difference could be due to difference in environment and socioeconomic conditions such as advancement in educational materials like computers. The finding was also in line with study results from Gondar town [12]. This finding is very lower than the finding from Cape Coast, Ghana in 2010 [13]. This variation could be due to the difference in time of the study, sample size differences or materials used for examination and methods of data collection.

In this study myopia (5.47%) was found to be the dominant cause of visual impairment in both right and left eye followed by astigmatism (1.9%) and hyperopia 1.4%. This finding was contradictory with a survey conducted in Iran where the finding showed astigmatism (11.5%), hyperopia (5.4%) and myopia (4.3%) irrespective of gender [14]. Similarly, our finding do not agree with the findings from kalaji which stated myopia to be common in boys [6]. This could be due to differences in race, time and socio economic set up.

The study revealed that a female was about 3.9 times more likely to experience refractive errors than male. This finding contradicts with the finding of the study conducted in Tafila city (Jordan) [15]. This difference might be due to differences in race, the time of the study and the method used. The prevalence of refractive errors in higher grade level (5–8) was about 4.8 times more likely than lower grade (1–4). This finding is similar with a study conducted in Iran and Malaysia [16,17] even though slight difference in number. This might be due to due to potential effect of sex on the problem.

In this study, the number of students who underwent preschool and school screening for refractive error was (5%), this is smaller when compared with finding of the study in conducted in Karachi Iran [6]. This could be due to the difference in awareness about importance of eye care, availability and accessibility to eye health care services.

Associated ocular morbidities in either eye were observed in 64 (15.2%) of the students. This finding was smaller than the findings from a study in Nepal [11]. This could be due to the difference in the study site (hospital based versus school based).

The uptake of refractive service was 4 (9.3%) and spectacle utilization was 1(4.6%). This uptake is very low compared with that of the findings from studies in Malaysia [18] and Singapore [19] where spectacle coverage was found to be 48% and 25% respectively. This disparity may come from different socio economic disparities, including infrastructure development or due to cultural differences, but it was found a bit higher when compared with the findings from Pakistan which stated the spectacle coverage at presentation [16].

The study also showed that using of computers regularly had increased the chance of having refractive error by 4.5% compared with irregular or non-users. This could be due to the effect of continuous light reflecting on eyes. This finding was also in line with the finding from Iran [6].

The limitations of this study was its not getting enough information about children’s family and its being institution-based than community-based. Moreover, we did not include rural children.

## Conclusion

The prevalence of uncorrected refractive error is high among Debre Markos District school children. Amblyopia was very high followed by hyperopia and astigmatism. Females were at higher risk than males. The uptake of refractive service as well as health education regarding refractive error was found to be very low. Thus, school health services should include eye screening. Health education should be given by giving special attention to the impact of refractive errors and spectacles utilization should be encouraged for the cases.

## Competing interests

The authors declare that they have no competing interests.

## Authors’ contributions

SA: conception and initiation of the study, design, implementation, analysis and writing. KK: conception and initiation of the study, design, implementation of the study and co-writing. MG: design, implementation of the study and co-writing. All authors read and approved the final manuscript.

## Pre-publication history

The pre-publication history for this paper can be accessed here:

http://www.biomedcentral.com/1471-2415/14/95/prepub
